# Virtual reality technology in the rehabilitation of post-stroke cognitive impairment: an opinion article on recent findings

**DOI:** 10.3389/fpsyg.2023.1271458

**Published:** 2023-10-02

**Authors:** Ting Zhang, Wei Liu, Qingping Bai, Song Gao

**Affiliations:** ^1^College of Physical Education and Health Sciences, Zhejiang Normal University, Jinhua, China; ^2^Department of Traditional Chinese Medicine, University Hospital, Zhejiang Normal University, Jinhua, China; ^3^Physical Education College, Guangxi University of Science and Technology, Liuzhou, China

**Keywords:** post-stroke, cognitive impairment, virtual reality technology, rehabilitation, limitations

## Introduction

Post-stroke cognitive impairment (PSCI) is a group of syndromes in which one or more cognitive dysfunctions, such as memory, executive function, orientation and attention, occur within 6 months after stroke (Lim et al., 2021). The prevalence of PSCI is as high as 64%, and 1/3 of these patients will progress to dementia, seriously affecting their quality of life (Delavaran et al., 2017). Post-stroke limb movement disorders have long received widespread attention and have a well-established clinical rehabilitation system (Nakawah and Lai, 2016). However, PSCI is often neglected.

Currently, oral medications and rehabilitation are the main treatment modalities for PSCI, but the therapeutic effects are not satisfactory. Animal models cannot reproduce several features of the pathogenesis of vascular cognitive impairment, which limits the development of relevant drugs (Kuang et al., 2021). To date, no drug for vascular cognitive impairment has been approved by the Food and Drug Administration (USA) (Gorelick et al., 2011). Vascular dementia and Alzheimer's disease (AD) overlap in their neuropathological mechanisms (Sun, 2018). Drugs for AD can also improve cognitive function and activities of daily living in patients with PSCI, such as donepezil, galantamine and memantine (Glass et al., 2020; Cichon et al., 2021). However, the side effects and potential harms of long-term drug use need to be considered (Shetty, 2013). Cognitive impairment is mainly assessed with paper and pencil tests (De Roeck et al., 2019). The occupational therapist provides therapeutic or adaptive tasks depending on the patient's cognitive status (Mijajlović et al., 2017). This type of training is tedious and the results vary widely in clinical practice. However, there is no clear treatment for PSCI (Rost and Brodtmann, 2022).

The effective integration of rehabilitation for cognitive impairment with computer technology has led to new and more interesting treatment modalities, such as virtual reality (VR) (Ge et al., 2018). VR is a new computer-generated technology that is being widely used clinically for the diagnosis, assessment, and treatment of mental disorders (Freeman et al., 2017). VR has two key features: immersion and interaction, providing a three-dimensional environment for the user to interact with the virtual environment through multiple sensory channels, including visual, auditory, and tactile (García-Betances et al., 2015). Previous studies have shown that cognitive training, physical training, and lifestyle can improve cognitive function (Stern, 2012; Huang et al., 2017). Compared to traditional rehabilitation therapies, VR incorporates some game-like elements that increase participants' motivation, cooperation, and satisfaction. VR can be designed with different scenarios and training to target different cognitive areas, and can be adapted in a timely manner according to patients' needs and cognitive status. In addition, compared to traditional paper-and-pencil tests, VR-based goal-directed navigation and recognition tasks are performed by immersing patients in a 3D virtual environment (La Corte et al., 2019). VR navigation tasks may involve more complex processes, including representational updating, spatial memory, and adaptation of the self when approaching a distant spatial target (Brument et al., 2021). These skills and manipulations cannot be accounted for in paper-and-pencil tests.

VR technology has been initially demonstrated in the treatment of cognitive disorders, such as memory impairment, attention impairment, executive function, language and numeracy impairment (D'Cunha et al., 2019). Several studies have shown that VR technology improves visuospatial motion perception, delayed memory, orientation and attention better than traditional cognitive rehabilitation (Liao et al., 2020; Torpil et al., 2021). Besides, a study by Liao et al. (2019) found significant improvements in self-care and gait in patients who underwent VR interventions (task-based interventions: simulated grocery store, simulated kitchen, finding a store, etc.). A meta-analysis found that VR was less effective in improving cognitive function in patients with AD than in normal elderly and people with MCI. However, the feasibility of VR technology has been demonstrated in a non-immersive VR intervention for patients with AD (Hofmann et al., 2003; Kim et al., 2019). Notably, the effect of the intervention was correlated with patient cooperation. Impaired memory and executive function are key features of AD. Several studies have used VR tasks to intervene with AD patients for 2–4 months (Serino et al., 2017; Oliveira et al., 2021). The results showed that the VR task was effective in improving patients' overall cognitive function, memory and executive function, especially spatial navigation and memory.

At present, with the rise of virtual reality technology, VR technology has a good prospect of being applied to PSCI. PSCI seriously threatens patients' quality of life, and VR technology has a potential role in rehabilitation. VR technology applied to the rehabilitation of PSCI not only allows patients to train in a safe and interesting environment, but also provides timely feedback on the effectiveness of treatment. This paper aims to outline the key role of VR technology in improving cognition in patients with PSCI. Moreover, the authors highlight the limitations and challenges of current research related to VR technologies for improving cognition in stroke patients.

## VR technology in PSCI rehabilitation

Cognitive function declines with age, particularly memory, executive function, processing speed, and reasoning, which are essential for maintaining the ability to perform activities of daily living (Konar et al., 2016). Therefore, it is recognized that delaying or preventing cognitive decline in healthy older adults is critical. Studies have shown that exercise and cognitive training can reduce cognitive decline in healthy older adults (Mondini et al., 2016; Feng et al., 2017). However, exercise and cognitive training have some shortcomings, such as lack of motivation, limitations of location and weather, and safety of going out. A study by Anderson-Hanley et al. (2012) combined VR with traditional physical exercise in healthy older adults and found that this method improved cognitive function and delayed the progression to mild cognitive impairment. Other studies have shown that VR improves executive function, attention and transfer performance in older adults, but is less effective in improving language and memory (Zajac-Lamparska et al., 2019; Sakaki et al., 2021).

Patients with PSCI have difficulty concentrating during rehabilitation training and get burned out when repeating the same training action multiple times. VR technology not only enhances the fun and initiative of rehabilitation but also allows for a variety of rehabilitation content according to the patients' needs. One study found significant improvements in memory, spatial and temporal orientation in patients using VR combined with computer-based training (Huang et al., 2019). A study of community-based PSCI patients found that VR-based cognitive training not only improved cognitive function but also improved patients' psychological status (Maier et al., 2020). The scenario content of VR cognitive training places progressively higher demands on patients' memory and attention skills (Gamito et al., 2017). The VR environment not only approximates the real environment, but can also allow the implementation of additional stimuli to present more information for assessment. Klinger and Marié simulated a medium-sized 3D supermarket with most of the items found in a real supermarket. It allowed participants to perform a shopping task: load a virtual shopping cart with different items from a predefined list, then place the items in the cart and pay at the checkout (Cogné et al., 2018). To address new questions, visual and auditory stimuli could later be added to the software according to research needs (Josman et al., 2014). A study by Cogné et al. (2018) found that non-contextual auditory stimuli in the Virtual Action Plan Supermarket could be used to train patients with executive dysfunction and to suppress disruptive stimuli.

Executive function is the cognitive and neural mechanisms activated by an individual to achieve a goal, including planning, working memory and impulse control of impulses (Laakso et al., 2019). A study by Rozental-Iluz et al. (2016) found that interactive video games can effectively stimulate cognitive co-movement and have the potential to improve executive function in stroke patients. A study by Rogers et al. (2019) concluded that an interactive tabletop virtual system using goal-directed and exploratory upper limb motor tasks could enhance neuroplasticity in patients. This VR system improves patients' cognitive executive skills and provides multimodal positive feedback, facilitating the recovery of cognitive-motor coupling. While playing the interactive video game, patients engage in low to moderate intensity physical activity. Physical activity increases neurotransmitter secretion in the brain, which improves proprioception, cerebral blood flow, and brain volume (Laitman and John, 2015). Exercise increases brain-derived neurotrophic factor, which promotes neuronal survival and prevents cognitive decompensation (Karssemeijer et al., 2017).

A study by Faria et al. (2016) used an urban environment simulated by Reh@City to train patients in activities of daily living. The results showed more significant improvements in overall cognitive, attentional and executive function in the VR group, whereas patients in the traditional training group only showed improvements in memory and social engagement. At a later follow-up, patients in the VR group showed more consistent improvements in cognitive function than those in the control group (Faria et al., 2020). Another virtual cognitive task based on Reh@Task maps patients' arm movements onto a virtual arm in a VR environment. Patients completed a series of cognitive exercises in the VR scenario using arm movements (Faria et al., 2018). The results suggest that VR, which combines cognition and movement, is more effective than conventional rehabilitation in improving patients' overall cognition.

## Limitations of VR technology in PSCI rehabilitation

VR technology combines visual and auditory feedback with motor training. It focuses the attention of patients with PSCI, helping to improve concentration and spatial awareness. Different scenarios and difficult tasks make the patient's movements more purposeful and directed. Repetitive training further consolidates the patient's thinking patterns and training effects. VR technology can improve walking ability, promote cognitive recovery, and improve activities of daily living in patients with PSCI (Xiao et al., 2022). However, there are still some problems with the application of VR technology: The sample size of existing studies is small. Multicenter clinical randomized controlled trials with large sample size should be added. Therefore, it is difficult to accurately assess the effectiveness of VR technology interventions at present; Elderly people may experience discomfort symptoms such as dizziness, nausea, and eye fatigue during VR training (Stoffregen et al., 2017; Kim et al., 2018). Therefore, how to overcome the adverse effects of VR needs further research; High-quality VR equipment is expensive, bulky, poorly mobile, and requires a large venue. Thus, these factors limit its widespread adoption to home and community use.

## Conclusion

PSCI recognition and subsequent treatment may be critical to the success of the overall rehabilitation process after stroke (Torrisi et al., 2021). In this paper, the authors showed that VR technology can improve cognitive impairment and increase engagement in patients with PSCI. VR technology provides multi-sensory stimulation for patients with PSCI. A more realistic experience in the virtual environment improves patients' somatic and cognitive functions. With the development of modern science and technology, VR technology will likely be more widely used in stroke rehabilitation, and it is use with PSCI holds much promise.

## Author contributions

TZ: Writing—original draft. WL: Writing—original draft. QB: Writing—review and editing. SG: Funding acquisition, Writing—review and editing.
